# Effect of Brain Computer Interface Training on Frontoparietal Network Function for Young People: A Functional Near‐Infrared Spectroscopy Study

**DOI:** 10.1111/cns.70400

**Published:** 2025-04-22

**Authors:** Yulan Xu, Yuan Lanhui Li, Guancong Yu, Zitong Ou, Shantong Yao, Yawen Li, Yuhong Huang, Jing Chen, Qian Ding

**Affiliations:** ^1^ Brain Function Monitoring and Modulation Lab, Department of Rehabilitation Medicine, Guangdong Provincial People's Hospital (Guangdong Academy of Medical Sciences) Southern Medical University Guangzhou Guangdong China; ^2^ International Department The Affiliated High School of South China Normal University Guangzhou Guangdong China; ^3^ Huamei Bond International College Guangzhou Guangdong China; ^4^ Guangdong Cardiovascular Institute Guangdong Academy of Medical Sciences Guangzhou Guangdong China; ^5^ School of Rehabilitation Medicine Shandong Second Medical University Weifang Shandong China

**Keywords:** attention, brain computer interface, functional connectivity, functional near‐infrared spectroscopy, motor imagery

## Abstract

**Aims:**

Inattention in young people is one of the main reasons for their declining learning ability. Frontoparietal networks (FPNs) are associated with attention and executive function. Brain computer interface (BCI) training has been applied in neurorehabilitation, but there is a lack of research on its application to cognition. This study aimed to investigate the effect of BCI on the attention network in healthy young adults.

**Methods:**

Twenty‐seven healthy people performed BCI training for 5 consecutive days. An attention network test (ANT) was performed at baseline and immediately after the fifth day of training and included simultaneous functional near‐infrared spectroscopy recording.

**Results:**

BCI performance improved significantly after BCI training (*p* = 0.005). The efficiencies of the alerting and executive control networks were enhanced after BCI training (*p* = 0.032 and 0.003, respectively). The functional connectivity in the bilateral prefrontal cortices and the right posterior parietal cortex increased significantly after BCI training (*p* < 0.05).

**Conclusion:**

Our findings suggested that repetitive BCI training could improve attention and induce lasting neuroplastic changes in FPNs. It might be a promising rehabilitative strategy for clinical populations with attention deficits. The right PPC may also be an effective target for neuromodulation in diseases with attention deficits.

## Graphical abstract

Our results suggested that repetitive BCI training could improve attention and induce lasting neuroplastic changes in FPNs. It might be a promising rehabilitative strategy for clinical populations with attention deficits.

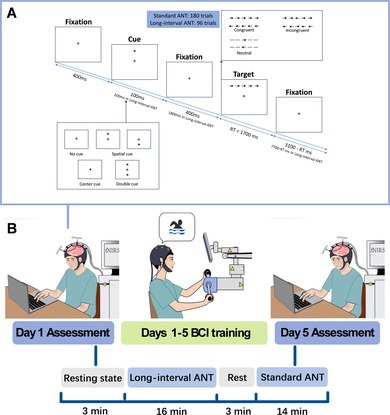

## 1 Introduction

Attention filters out irrelevant information and enhances the ability to process important information [1]. The human attention system is divided into three distinct functions: alerting, orienting, and executive control [2]. Executive functions play a crucial role in attention, decision making, and complex conflict processing [3, 4]. The efficiency of the attention network is usually quantified by an attention network test (ANT) [5]. The ANT simultaneously detects the function of three networks in a single test by calculating from the combinations of different cue conditions and stimuli. This test has been employed extensively in assessing attention disorders [6, 7, 8].

Inattentiveness and ineffective decision making are major sources of impaired learning efficiency for young people. Additionally, executive functions are involved in word reading and reading fluency. Executive dysfunction is generally believed to be a potential contributor to inattention and reading disability [9, 10]. Therefore, executive function impairments and inattentiveness may be key mediating effects in learning problems in young people. The neural mechanisms underlying inattentiveness, ineffective decision making, and executive dysfunction remain unclear but have been suggested to relate to frontoparietal networks (FPNs), including mainly the prefrontal cortex (PFC) and posterior parietal cortex (PPC) [11]. A previous study reported that young people with inattentiveness showed significantly reduced resting‐state functional connectivity within the FPNs [11]. As a result, increasing the functioning of FPNs could be a possible strategy for young people with inattentiveness or ineffective decision‐making. Further research is needed to explore the specific mechanisms of how the training programs improve attention.

Brain computer interface (BCI) is a technology that translates brain activity into motor commands for controlling external devices [12, 13]. BCI training, which has been widely used in neurorehabilitation, is a training program that commonly combines BCI and motor imagery training [14]. Several studies have demonstrated the potential of BCI training to facilitate the rehabilitation of motor function in patients with movement disorders [15, 16, 17]. Improvements in motor function after a stroke are based on the neuroplasticity induced by BCI [12, 18]. Recent studies have reported that BCI not only facilitates the recovery of motor function but also improves attention in stroke patients [19]. BCI training can also effectively improve attention in children with attention‐deficit/hyperactivity disorder (ADHD) [20]. However, brain function was not monitored in the above studies. Therefore, the neural mechanisms underlying BCI training to improve attention remain unclear. An fMRI study reported that the improved attention after motor imagery training was accompanied by increased FPNs functioning [21], suggesting that modulations of FPNs functions could be a possible mechanism underlying the improved attention after BCI training. This needs to be further investigated.

BCI training involves complex cognitive processes requiring the activation of multiple cortical areas, such as the prefrontal cortex and parietal cortex [22]. In addition to cortical activation, network efficiency and functional connectivity are new perspectives to investigate the interrelation in different cortices and changes in brain networks after BCI training. Many studies have shown that BCI induces enhanced connectivity in attention‐related pathways [23, 24, 25]. Thus, investigating cortical activation and functional connectivity after BCI training could provide insights into neural mechanisms underlying the effects of BCI training on attention. Functional near‐infrared spectroscopy (fNIRS) is a noninvasive tool that has been widely employed to assess cortical activation and functional connectivity in multiple cortical regions simultaneously [12]. The fNIRS has been used to investigate changes in brain activities after BCI training [12, 26], but brain activities have not been measured during ANT. Increased brain activation [26, 27, 28, 29] and functional connectivity [23, 30] in parietal and prefrontal cortices have been reported after BCI training, but most studies have focused on the recovery of motor function. Therefore, BCI training has the potential to improve attention, possibly by increasing brain activation and functional connectivity. However, this needs further investigation.

In this study, we investigated the effects of BCI training on attention network functions in healthy young people. Our hypotheses were the following: (1) the efficiency of the attention network will be enhanced after BCI training; (2) brain activation in the parietal and prefrontal cortices during ANT will be improved after BCI training; and (3) functional connectivity in the parietal and prefrontal cortices during ANT will be improved after BCI training.

## 2 Methods

### 2.1 Participants

A total of 27 healthy young people, 12 males and 15 females with a mean age of 23.0 years (SD = 1.6), participated in this study. The sample size was determined by power analysis using G‐Power software (version 3.1). The participants were assessed as right‐handed in accordance with the Edinburgh Handedness Inventory and had no history of neurological disorders, including head or hand injuries. Prior to commencing the experiment, participants were required to provide written informed consent. This study was approved by the Guangdong Provincial People's Hospital Human Research Ethics Committee (KY2023‐1079‐02) and has been registered at the Chinese Clinical Trial Registry (ChiCTR2500097678). This study was conducted in accordance with the Declaration of Helsinki.

### 2.2 Clinical Assessment

Clinical assessments, including the kinesthetic and visual imagery questionnaire (KVIQ‐20) and intrinsic motivation inventory (IMI), were performed on the first and fifth days of the experiment. The KVIQ‐20 is a 20‐item assessment that uses a five‐point scale to evaluate the clarity of images and the intensity of sensations, which are used to assess the visual and kinesthetic aspects of motor imagery [31]. The intrinsic motivation inventory (IMI) has two questionnaires randomly generated from four sessions of 37 questions each to explore the influence of intrinsic motivation. The overall assessment unfolds from these four subjective experience dimensions: interest/enjoyment, perceived competence, perceived choice, and pressure/tension [32].

### 2.3 Attention Network Test

**FIGURE 1:**
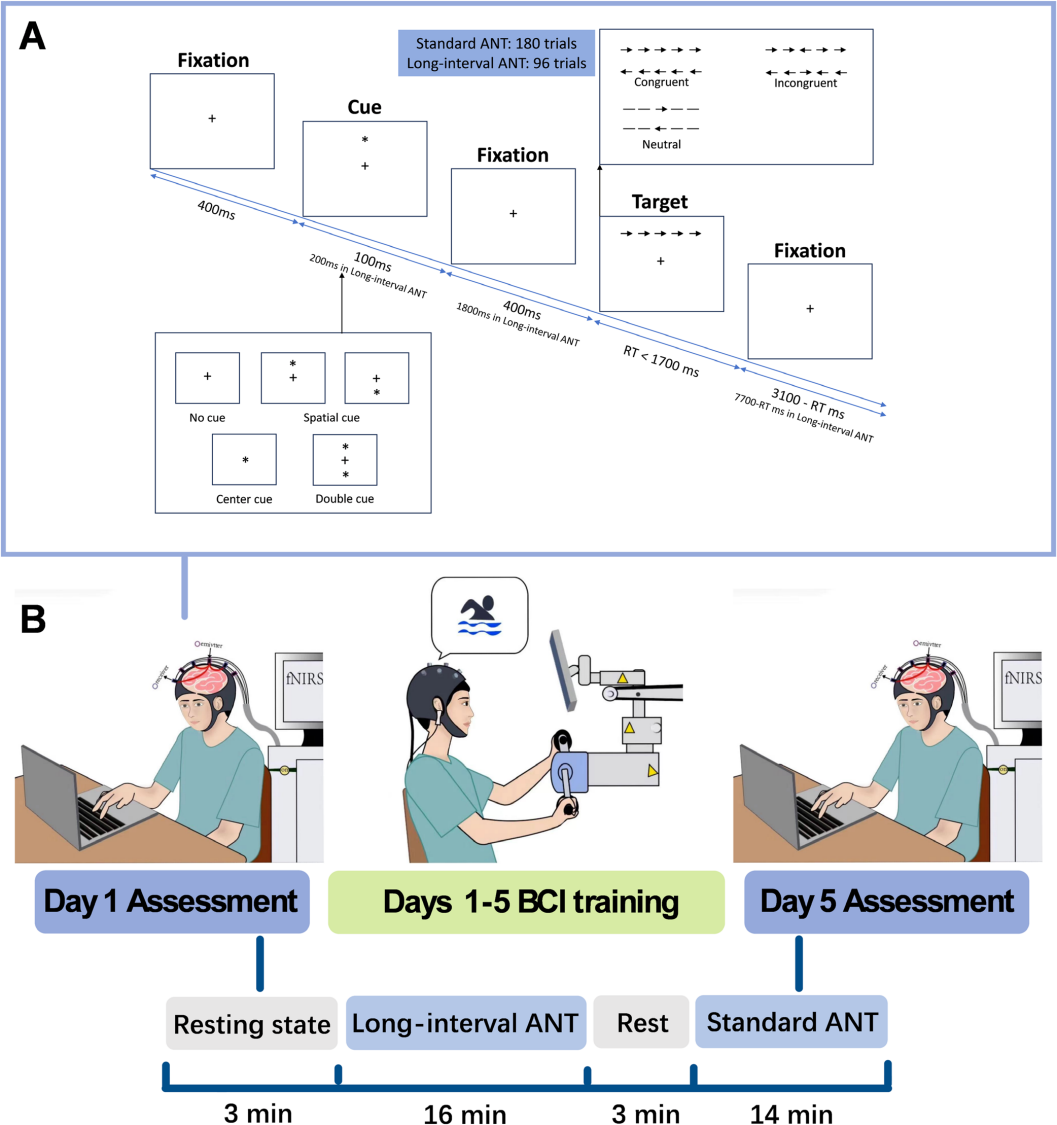
Detailed procedure of experiment. (A) Schematic of the attention network test. At the beginning of each trial, a fixation point lasting 400 ms was presented in the center of the screen. This was followed by a cue stimulus that lasted 100 ms in the standard ANT (200 ms in the long‐interval ANT). Then, the fixation reappeared (lasting 400 ms in standard ANT and 1800 ms in long‐interval ANT). After that, the target stimulus was presented, and the subject had a maximum reaction time (RT) of 1700 ms. Fixation points appeared immediately after the response, and their duration correlated with the length of the RT. (B) Experimental procedure. Subjects underwent BCI training for 5 days. The fNIRS and ANT assessments were conducted at baseline and after BCI training. During the fNIRS assessment, subjects stayed at rest for 3 min and then performed long‐interval ANT and standard ANT with a 3‐min rest break between the two tasks.

Participants were seated in a quiet room, their eyes 65 cm away from a laptop computer screen. E‐prime 3.0 experiment software was applied to program the ANT. To ensure the blood oxygen levels fell back to baseline [33], we decided to use a special version of ANT with a longer inter‐trial interval (i.e., long‐interval ANT). There were 291 rounds of experiments divided into three stages, including 15 trials of practice, 96 trials of long‐interval ANT, and 180 trials of standard ANT (60 trials a round with three repetitions and two 1‐min rests). The total duration was 30 min. Between the long‐interval ANT and standard ANT, there was a rest for about 3 min to reduce the influence of fatigue. Each trial consisted of five steps [5]. Details see Data S1. The detailed procedure of ANT is illustrated in Figure 1A.

According to Fan et al. [5], the effects of the alerting, orienting, and executive control networks were defined as RT differences. For every participant, the mean RTs were calculated for all 12 combinations of the four cue conditions (no, central, double, spatial) and the three flanker conditions (congruent, incongruent, neutral).

$${\text{Efficiency}}_{\left(\text{alerting}\right)}={\mathrm{RT}}_{\left(\mathrm{no}\;\mathrm{cue}\right)}-{\mathrm{RT}}_{\left(\text{double}\;\mathrm{cue}\right)}$$

$${\text{Efficiency}}_{\left(\text{orienting}\right)}={\mathrm{RT}}_{\left(\text{central}\;\mathrm{cue}\right)}-{\mathrm{RT}}_{\left(\text{spatial}\;\mathrm{cue}\right)}$$

$${\text{Efficiency}}_{\left(\text{executive}\right)}={\mathrm{RT}}_{\left(\text{incongruent}\right)}-{\mathrm{RT}}_{\left(\text{congruent}\right)}$$

The selected trials contained only neutral and congruent conditions when the efficiencies of the alerting and orienting networks were calculated. All the trials used for calculations were correct, and their RTs were longer than 150 ms and less than 1000 ms. The longer RT of the alerting network and the orienting network and the shorter RT of the executive control network indicated higher efficiency.

### 2.4 fNIRS

#### 2.4.1 fNIRS Acquisition

**FIGURE 2:**
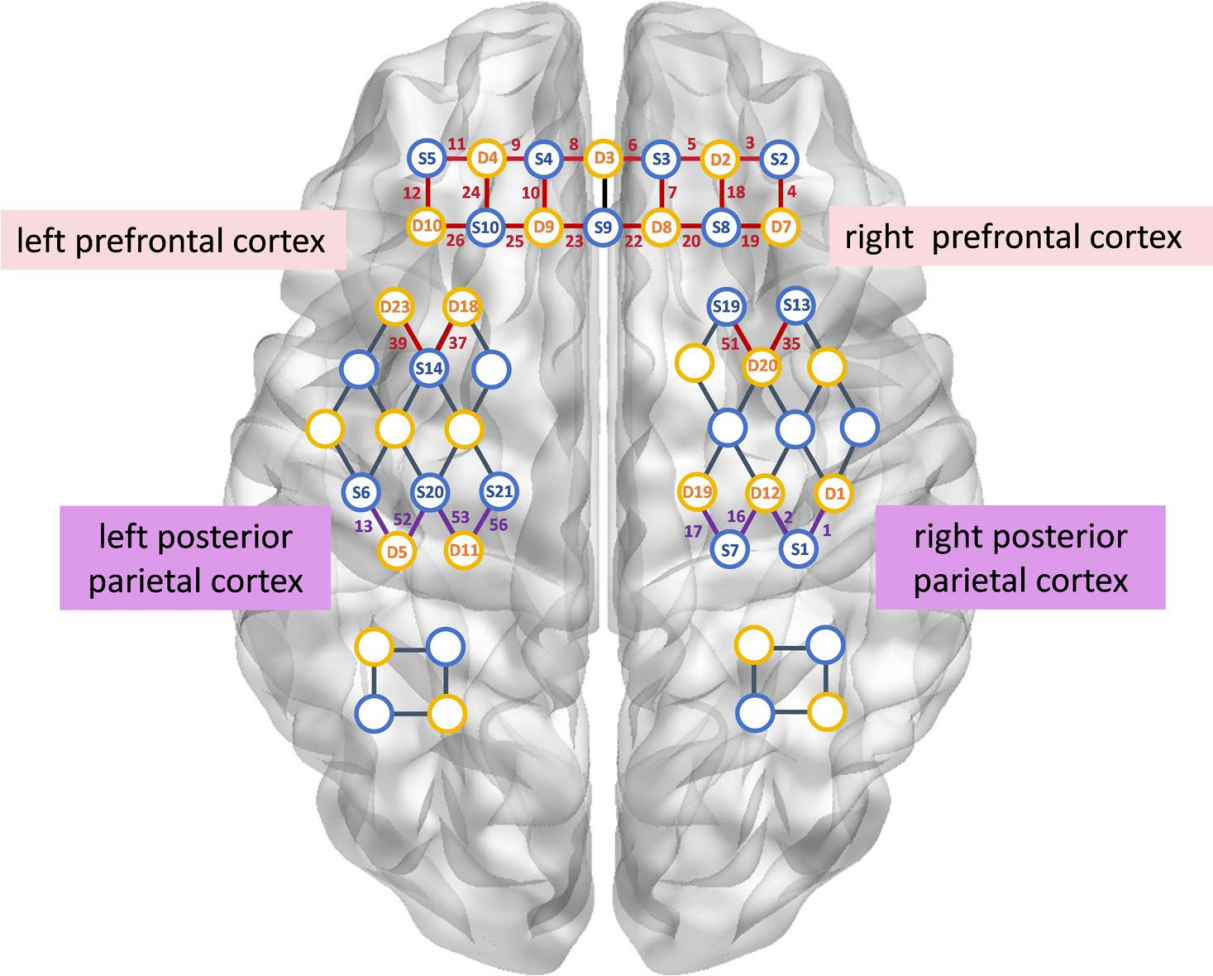
Distribution of fNIRS channels. The blue circles represent the source, and the yellow circles represent the detectors. The lines between the blue and yellow circles indicate the channels, and the number represents the channel number. The fNIRS channels are distributed in the bilateral parietal and bilateral prefrontal cortices.

In this experiment, a 63‐channel (24 emitters and 24 receivers) fNIRS device (NirScan, Danyang Huichuang Medical Equipment Co. Inc., Jiangsu, China) was employed to detect changes in cerebral oxy‐Hb and deoxy‐Hb concentrations during ANT tasks and resting state [34, 35]. Three wavelengths of near‐infrared light (730, 808, and 850 nm) were used. The distribution of channels is illustrated in Figure 2.

Regions of interest (ROIs) were selected via Polhemus PATRIOT digitizer channel registration analyses. Details see Data S1. The selected ROIs were the left PPC (Channels 13, 52, 53, 56), right PPC (Channels 1, 2, 16, 17), left PFC (Channels 8–12, 23–26, 37, 39), and right PFC (Channels 3–7, 18–20, 22, 35, 51). All channels with > 50% overlap within a region were averaged together based on MRIcro registration [36, 37]. The sampling frequency was set at 11 Hz. Prior to the commencement of data acquisition, a series of gain quality checks were conducted to ensure that neither under‐gain nor over‐gain was observed in the data.

#### 2.4.2 fNIRS Analysis

The analysis of fNIRS signals was performed using matlab2019a (Mathworks Inc.). fNIRS data were preprocessed with MATLAB scripts and Homer2 toolbox. MATLAB scripts were used to calculate the brain activation level and functional connectivity. Coherence was calculated, indicating functional connectivity. The GRaph thEoreTical Network Analysis (GRETNA) toolbox was used for graph theory analysis. The nodal efficiency and degree centrality were calculated. Details see Data S1.

### 2.5 BCI Training

An EEG‐driven BCI rehabilitation training equipment (Xiangtan Mailian Medical Equipment Co. Inc., Hunan, China) was used in the current study, which integrates EEG acquisition and motor training [38, 39]. The position of the conductive electrode was arranged in accordance with the international 10–20 rule. The electrode cap was connected to the amplifier device via a lead wire. The motor proportion was recorded at the end of training for qualifying the training effect. Details see Data S1.

### 2.6 Experimental Procedure

The baseline assessment was conducted on the first day, and BCI training was continued from Days 1 to 5. Each BCI training session lasted for 10 min and was of moderate intensity. The behavioral data of ANT and functional near‐infrared brain imaging were collected before the first day and after the fifth day of BCI training (Figure 1B).

### 2.7 Statistical Analysis

Statistical analysis was performed using IBM SPSS Statistics 27. Data were found to meet the normality assumption using the Shapiro–Wilk test.

A paired *t*‐test model was employed to evaluate the changes in alerting, orienting, and executive network efficiency of ANT before and after BCI training.

Repeated‐measure ANOVA models were applied to compare motor imagery ability [Day (2) × Type (2)], intrinsic motivation [Day (2) × Dimension (4)], brain activation [Day (2) × ROI (4)], coherence of six types of connectivity [Day (2) × ROI (6)], nodal efficiency and degree centrality [Day (2) × ROI (4)] before and after BCI training. Bonferroni corrections were used for multiple comparisons for all ANOVA models.

For all analyses, the statistical significance was set at *p* < 0.05, and adjusted *p* values are presented.

## 3 Results

### 3.1 BCI Performance

**FIGURE 3:**
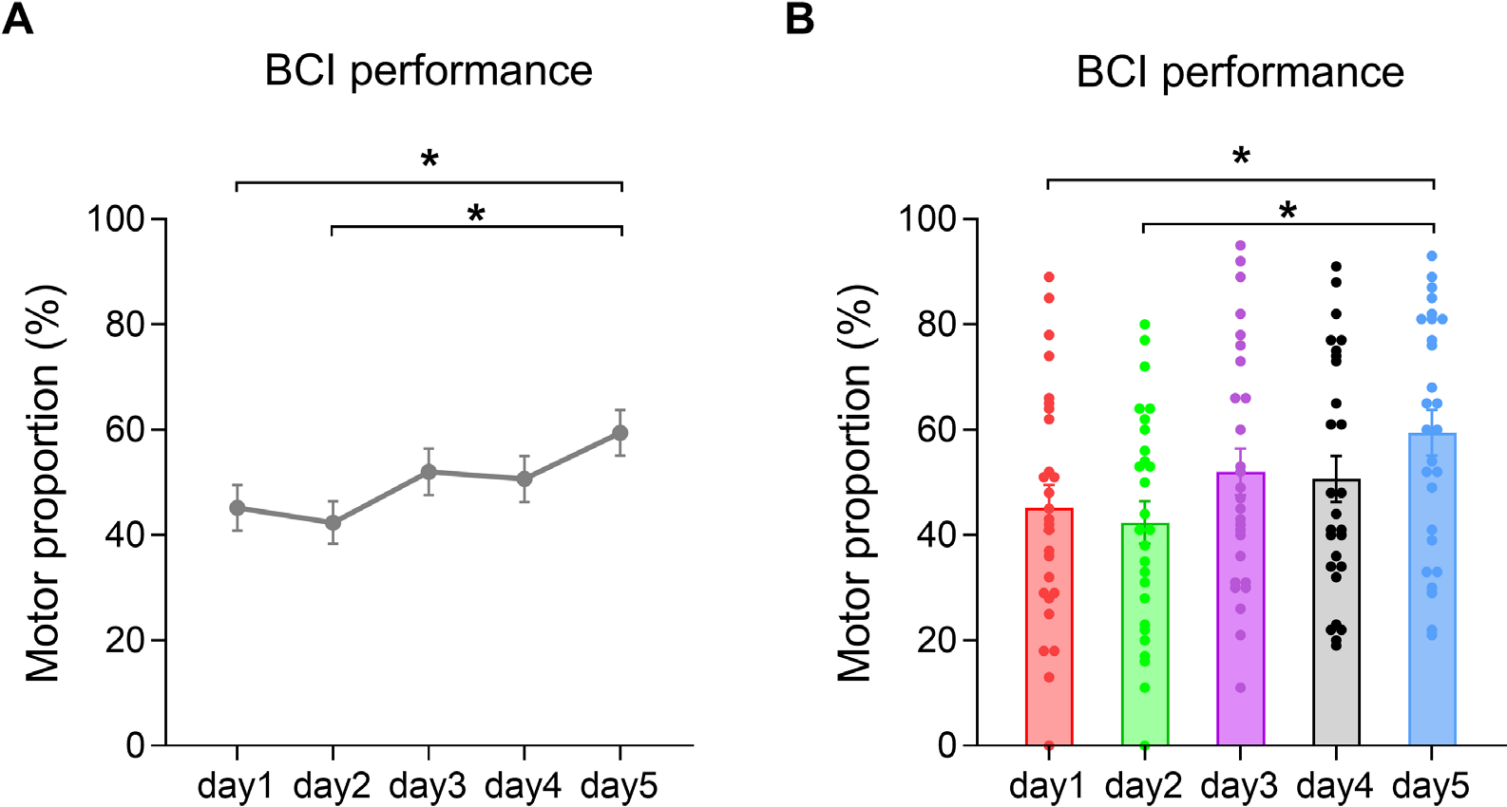
The motor proportion of BCI. The motor proportion on the fifth day increased significantly compared with the first and second days. *p < 0.05.

The ANOVA model revealed a significant main effect of Day [*F*_(3.29,85.53)_ = 4.389, *p* = 0.005]. Post hoc results showed that motor proportion was increased significantly on the fifth day compared with the first (*p* = 0.035) and second days (*p* = 0.017) (Figure 3).

### 3.2 Clinical Assessment

For KVIQ‐20, the ANOVA model revealed a significant main effect of Day [*F*_(1,26)_ = 6.859, *p* = 0.015] (Figure S1). For intrinsic motivation, the ANOVA model did not reveal a significant main effect [*F*_(1,26)_ = 1.362, *p* = 0.254].

### 3.3 ANT

**FIGURE 4:**
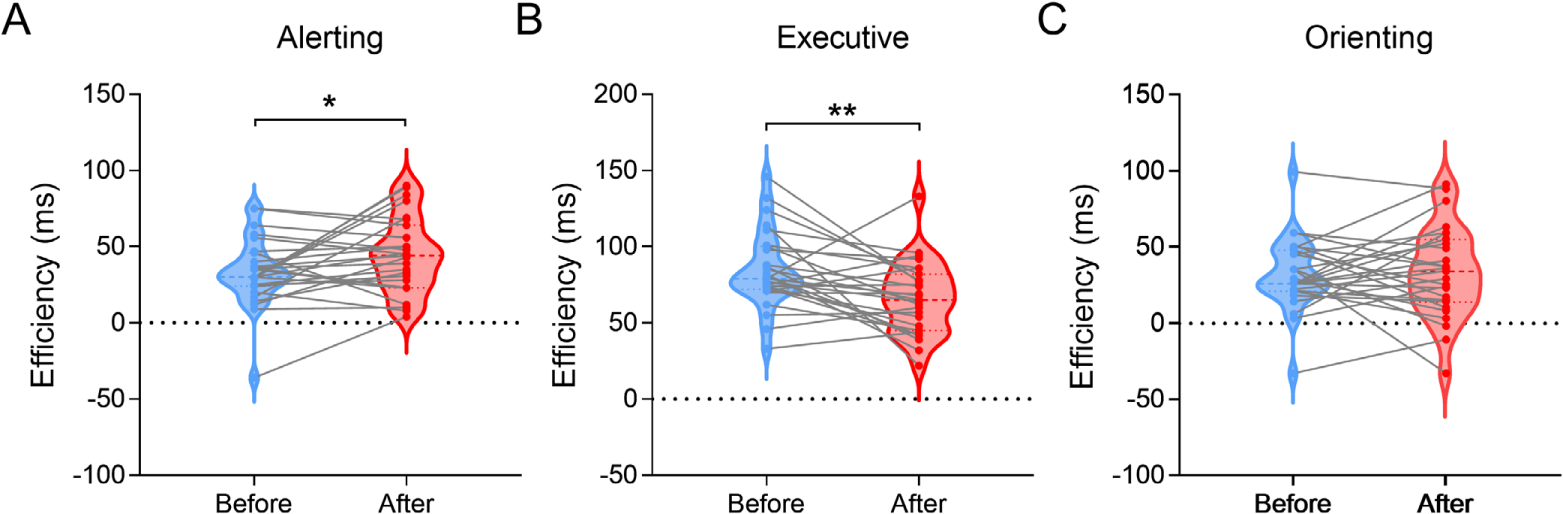
Efficiency changes in attention networks after BCI training. (A) After BCI training, the efficiency of the alerting network improved significantly. (B) The efficiency of the executive control network was significantly improved. (C) The efficiency of the orienting network was not significantly changed. *p < 0.05; **p < 0.01.

The paired *t*‐test revealed significant effects on the alerting network (*t* = 2.265, *p* = 0.032) and executive control network (*t* = −3.223, *p* = 0.003) after BCI training. There was no significant difference in the orienting network after BCI training (*p* = 0.812). The efficiencies in executive control and alerting increased (Figure 4).

### 3.4 fNIRS Data

#### 3.4.1 Brain Activation

The ANOVA model revealed no significant difference in brain activation in the alerting, orienting, and executive control networks from before to after BCI training [*F*_(1,26)_ = 0.318, *p* = 0.578; *F*_(1,26)_ = 1.659, *p* = 0.209; *F*_(1,26)_ = 0.150, *p* = 0.702, respectively].

#### 3.4.2 Functional Connectivity

**FIGURE 5:**
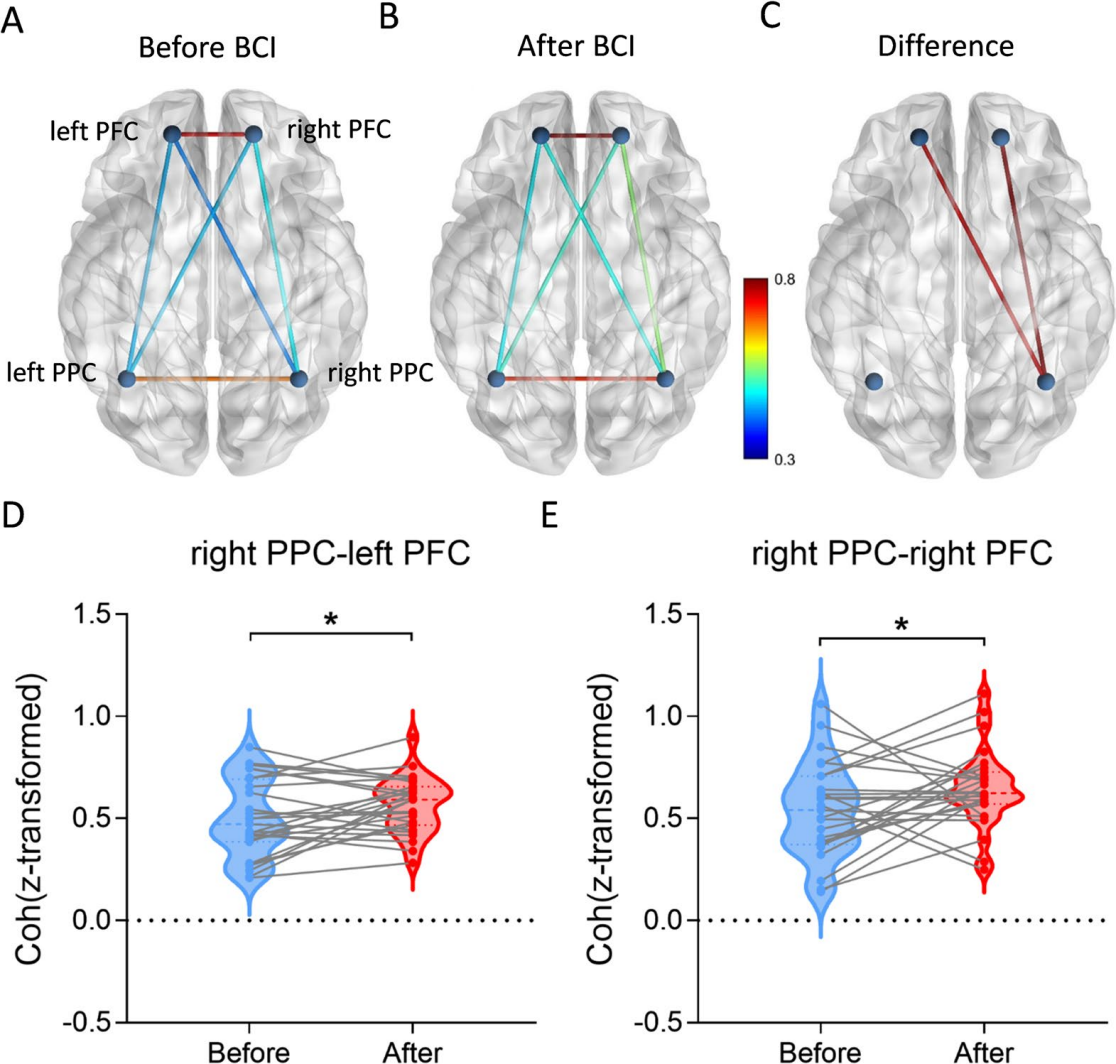
Functional connectivity among bilateral prefrontal and bilateral parietal cortices. (A) Coherence before BCI training. (B) Coherence after BCI training. (C) Coherence between bilateral PFC and right PPC was increased significantly after BCI training. (D) Coherence between the right PPC and left PFC was significantly increased after BCI training. (E) Coherence between the right PPC and right PFC was significantly increased after BCI training. *p < 0.05.

For resting‐state functional connectivity, the ANOVA model revealed no significant changes as a result of BCI training [*F*_(1,26)_ = 0.226, *p* = 0.639]. For task‐dependent functional connectivity, the ANOVA model revealed significant main effects of ROI [*F*_(1,26)_ = 53.032, *p* < 0.001] and Day [*F*_(1,26)_ = 5.906, *p* = 0.022] but revealed no significant effect of ROI × Day interaction [*F*_(1,26)_ = 0.528, *p* = 0.753]. Post hoc analysis showed that coherence between the left PFC and right PPC (*p* = 0.042) and between RPFC and right PPC (*p* = 0.049) was enhanced during the normal ANT after BCI training (Figure 5).

#### 3.4.3 Network Efficiency

**FIGURE 6:**
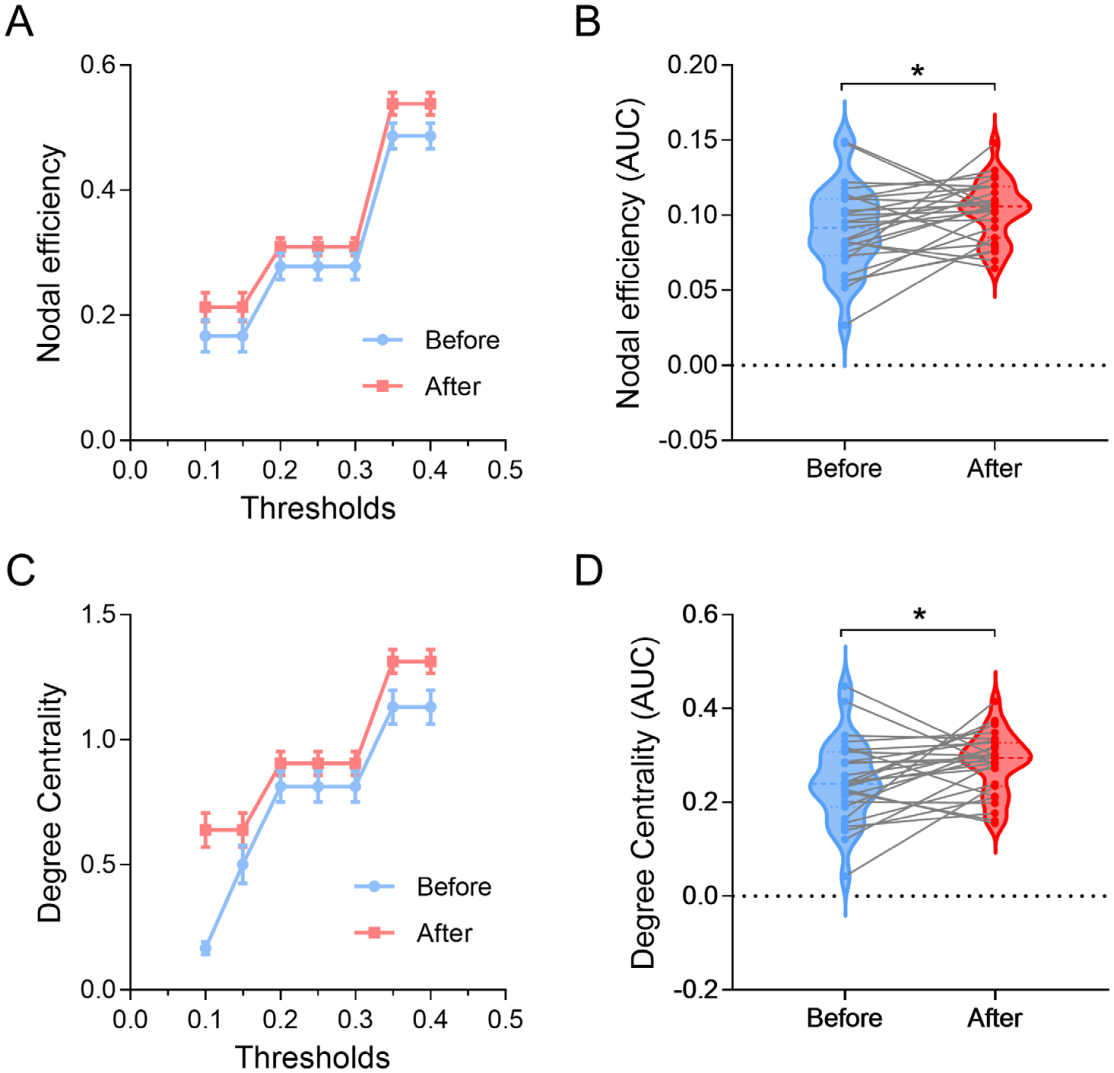
Nodal efficiency and degree centrality in the right PPC. Nodal efficiency (A) and degree centrality (C) at each threshold before and after BCI training. Nodal efficiency (B) and degree centrality (D) were significantly increased after BCI training. The AUC means the area under the curve. *p < 0.05.

The ANOVA model revealed no significant effect of Day for nodal efficiency [*F*_(1,26)_ = 3.042, *p* = 0.093]. Post hoc analysis showed that nodal efficiency was significantly increased in the right PPC after BCI training (*p* = 0.038). The ANOVA model revealed no significant effect of Day for degree centrality [*F*_(1,26)_ = 4.147, *p* = 0.052]. Post hoc analysis showed that degree centrality was significantly increased in the right PPC after BCI training (*p* = 0.049) (Figure 6).

## 4 Discussion

In this study, we presented the effects of BCI training on the functioning of FPNs, including the PPC and PFC, in healthy young people. In addition, we monitored brain activation status and calculated brain network indicators such as functional connectivity, which enabled us to investigate the potential mechanism of BCI training on FPNs from the perspective of brain function. The main findings of the present study were the following: (1) the ability of BCI control improved significantly; (2) The efficiencies of the alerting network and executive control network were enhanced after BCI training; (3) increased functional connectivity was observed in the bilateral prefrontal cortices and the right PPC; and (4) the brain network efficiency in the right PPC was significantly enhanced. In summary, the results were consistent with our expectations. Both the ANT and brain function results indicated that BCI training could improve attention network function. Compared with previous studies, our study was the first to use fNIRS to monitor brain function changes during ANT, providing new evidence that BCI training improved attention performance.

### 4.1 BCI Performance Was Significantly Improved After BCI Training

BCI performance was significantly improved on the fifth day compared with the first day. This suggested an improved ability to control the BCI device, which has been suggested to be related to several factors, such as attention, motor imagery, and psychological engagement [40]. As all of our participants were BCI‐naive users, controlling the BCI device was a new and challenging task for them, indicated by the relatively low motor proportion score on the first day of BCI training. Consequently, they needed to continuously adjust their strategies for BCI control during repetitive BCI training sessions and gradually developed the skills to control the BCI device. As the participants were healthy young people without motor imagery problems, one of the strategies for improving BCI control ability was increased attention; that is, the ability to maintain concentration during BCI training based on the external feedback provided by the BCI device. An improvement in motor imagery ability was also reflected in the KVIQ‐20. Repeated motor imagery and motor execution formed a feedback loop in which attention is continuously improved. The IMI results suggested that there was no change in the intrinsic motivation of the subjects, indicating that the effect generated by BCI training was objective and not related to the subjects' own state. The above behavioral improvement likely resulted from experience‐dependent neuroplastic changes in the cognitive‐motor network induced by repetitive training of challenging tasks [41]. This will be discussed in the following sections in detail.

### 4.2 The Efficiency of Alerting Network and Executive Control Network Was Significantly Improved After BCI Training

The change in ANT serves as a behavioral indicator for evaluating attention network efficiency. After BCI training, there was a significant enhancement in the efficiencies of the alerting and executive control networks. Our findings were in line with a previous study [19] and indicated that BCI training positively impacted attention. This might be due to neurofeedback training via BCI enhancing cognitive performance [42]. Furthermore, repetitive training processes can assist subjects in improving their attention level [19, 43] and promote lasting neuroplasticity through repetitive movements and cognitive engagement. Consequently, this can enhance cognitive function and attention status [44]. Attention to motor control during training programs may be considered a potential mechanism through which specific components of executive control in young adults could be enhanced by altering a speed‐accuracy tradeoff [45]. Based on this feedback, participants assess their current state of motor imagery and adjust their attention concentration accordingly. We believe that decision making triggered in this manner is one reason why BCI training enhanced participants' executive function [46].

We hypothesized that increased efficiency would also be observed in the orienting network. However, contrary to previous studies [19], no significant change in the orienting network was observed here. We currently lack an explanation for this phenomenon. We compared the differences with previous studies and concluded that possible reasons for this phenomenon were the lack of training time or the effect of BCI on healthy people was not as obvious as in stroke patients.

### 4.3 Functional Connectivity Was Increased in Prefrontal and Parietal Cortices After BCI Training

The analysis of functional connectivity revealed a significant enhancement in coherence between the bilateral prefrontal cortices and right PPC. Furthermore, there was a significant improvement in network efficiency after BCI training. Research has demonstrated that the neuroplasticity of motor imagery‐related cortical networks (including sensorimotor cortex and PFC) is heightened after BCI training [12], which may induce improvements in the ability of motor imagery and BCI control. In an fMRI study, functional connectivity between the thalamus and PFC and cerebellum was strengthened after 10 days of BCI training [24]. These regions are components of the cerebellar–thalamocortical pathway, which plays a role in executive functions [47]. Additionally, connectivity between the thalamus and PFC (especially DLPFC) is linked to attention processing [24]. Therefore, we hypothesized that BCI enhanced behavioral performance by regulating brain functional connectivity associated with processes of attention and executive control. The DLPFC has connections with visual areas of the inferior parietal cortex and is involved in visual and auditory top‐down attention [30], consistent with improved coherence between the left PFC and right PPC after BCI training. It can be seen that the mechanism by which BCI training improved attention might be by regulating the connectivity of the above neural pathways. Interestingly, our results involved increased functional connectivity between the hemispheres. Similar findings have been observed in BCI training based on bimanual coordination motor imagery, showing enhanced connections between the parietal cortex and bilateral prefrontal cortices [23]. Several recent studies have indicated varying degrees of enhancement in interhemispheric functional connectivity following BCI intervention in stroke patients [25, 48, 49, 50]. Our results and these studies suggested that BCI modulated interhemispheric functional connectivity, which may be a mechanism through which it improved cognitive function.

Furthermore, we also observed enhancements in the efficiency of brain networks after BCI training. This is consistent with Xie's study demonstrating that motor imagery rehabilitation training significantly enhanced the efficiency of brain networks in processing motor‐related information, which was achieved by promoting effective redistribution of brain resources and enhancing interregional activities during motor imagery [51]. It can be seen that improvements in network efficiency and functional connectivity could contribute to the improvements in attention functions.

### 4.4 No Significant Change in Brain Activation Was Revealed After BCI Training

We did not observe significant changes in brain activation during ANT after BCI training. Neuroimaging studies have suggested that motor imagery training consistently recruits networks in bilateral premotor areas (supplementary motor areas, dorsal and ventral premotor cortex, cingulate gyrus, and putamina), cephalic inferior and medio‐superior parietal lobes (inferior and superior parietal lobule), basal ganglia, and cerebellar regions [22, 52, 53], indicating that BCI training could activate the attention‐related brain networks. In the current study, increased ANT performance, unchanged brain activation, and increased functional connectivity in FPNs after BCI training suggested that enhanced FPNs efficiency, rather than brain activation, likely contributed to the increased behavioral performance after BCI training.

### 4.5 Clinical Implications

Our study investigated the effects of BCI training on attention networks in healthy young people. We observed that the functions of the three attention networks were improved after BCI training, accompanied by an increase in network efficiency and functional connectivity between the right PPC and bilateral prefrontal cortices. This suggested that 5 days of BCI training could improve attention functions, accompanied by lasting neuroplastic changes manifested by increased FPNs functioning. Our findings have implications for both healthy people and clinical populations.

The BCI device used in the current study was noninvasive and safe. This provided BCI training with the potential to be utilized by healthy people in their daily lives. For young people, attention networks were suggested to be important for learning abilities of courses involved in intelligence [54]. For the elderly, they gradually show a physiological decline in memory, attention, and other cognitive functions with age [55]. Therefore, BCI training provides an incentive to develop better and less time‐consuming ways to improve FPN functions for improving learning ability of young people and daily life of the elderly.

Our study also provided a theoretical basis for improving attention and executive functions with BCI training in various neurological disorders, such as ADHD and stroke. BCI training provides a feedback loop to improve the inattention symptoms for ADHD patients. For stroke patients, most neurorehabilitation studies have focused on motor recovery [56, 57]. In fact, the enduring impact of post‐stroke cognitive impairment, encompassing symptoms of attention and executive dysfunction, surpasses that caused by physical dysfunction [58]. BCI training could serve as a novel approach to improve FPNs functions for stroke patients with cognitive impairment. The rehabilitative effect and neural mechanisms of BCI training on diseases with attention deficits need further exploration.

Unlike previous studies highlighting the importance of the PFC on attention and executive functions [59, 60], our results of functional connectivity and network efficiency implied that the right PPC played an important role in the attention network. BCI training effectively enhanced local efficiency in the right PPC, leading to increased functional connectivity between the right PPC and attention‐related regions to achieve improvements in FPNs. Consistent with previous studies reporting a strong relationship between the PPC and attention modulation [61, 62], the findings of the current study provided further support for the important role that the right PPC plays in attention. Therefore, the PPC can be utilized as an effective target for neuromodulation in diseases with attention deficits.

### 4.6 Limitations

First, our participants were young and highly educated people. In the future, we need to test our results in other populations and use larger sample sizes. Second, our current design did not include sham stimulus conditions, which may weaken the persuasiveness of the conclusion. Future experiments with sham stimulus conditions need to be completed.

## 5 Conclusions

Our study investigated the effects of BCI training on attention networks in healthy young people. Our results showed that the function of the three attention networks improved after BCI training and was accompanied by increased functional connectivity in the right PPC and bilateral PFC. These findings suggested that repetitive BCI training could induce lasting neuroplastic changes in FPNs, and the right PPC played an important role in attention. BCI training has the potential to improve learning abilities in young people by enhancing attention and may be utilized as a promising rehabilitative strategy for clinical populations with attention deficits. The right PPC may also serve as an effective target for neuromodulation in diseases with attention deficits.

## Author Contributions

**Yulan Xu:** writing – review and editing, writing – original draft, visualization, software, methodology, investigation, formal analysis, data curation. **Yuan Lanhui Li:** writing – review and editing, methodology, investigation, formal analysis, data curation. **Guancong Yu:** writing – review and editing, methodology, investigation, formal analysis, data curation. **Zitong Ou:** methodology, investigation. **Shantong Yao:** methodology, investigation. **Yawen Li:** methodology, investigation. **Yuhong Huang:** supervision, resources, project administration, conceptualization. **Jing Chen:** supervision, resources, project administration, conceptualization. **Qian Ding:** writing – review and editing, resources, project administration, funding acquisition, conceptualization.

## Conflicts of Interest

The authors declare no conflicts of interest.

## Supporting information


Data S1.

## Data Availability

The data that support the findings of this study are available from the corresponding author upon reasonable request.
