# Supplementary material for: Effect of Brain Computer Interface Training on Frontoparietal Network Function for Young People: A Functional Near‐Infrared Spectroscopy Study
Source: CNS Neurosci Ther. 2025 Apr 22;31(4):e70400. doi: 10.1111/cns.70400 (PMC12012575; doi:10.1111/cns.70400)
Supplement: Supplementary file 1 — Data S1. [file CNS-31-e70400-s001.docx]

**Supplemental information**

**Detailed procedure of ANT**

First, there was a fixation period, a central fixation cross that was displayed for 400ms. Then, a cue was presented for 200ms in long ANT and 100ms in standard ANT. After the cue signal, there was a short fixation period of 400ms in standard ANT and 1,800ms in long-interval ANT. Participants were required to respond to the target arrow direction by pressing the keyboard F to the left and J to the right for no more than 1,700ms. After participants made a response, the target and flankers disappeared immediately and there was a post-target fixation period for a variable duration, which was based on the duration of the reaction time (RT) [3500ms minus the duration of the first fixation (400ms in standard ANT and 1,800ms in long-interval ANT) minus RT]. Each trial lasted 4,000ms in standard ANT and 10,100ms in long-interval ANT. The central fixation point remained in the center of the screen throughout the experiment.

**Detailed procedure of selecting regions of interest**

Regions of interest (ROIs) were selected via Polhemus PATRIOT digitizer channel registration analyses. After the tasks were completed, the subjects were instructed to keep the fNIRS cap on while the experimenters carefully removed the optodes. A measuring tape was used to find the center point (i.e., Cz) on the head. Measurements were taken from the left auricular lobule to the right auricular lobule and from the nasion to the inion. Once the Cz point was determined, a magnet was positioned on it, and the subject was moved so that the inion was 10 cm away from the transmitter. Five head base reference points (nasion, left tragus, right tragus, inion, and Cz) were measured using the stylus. All other optical fiber points were measured in numerical order afterward.

**Detailed procedure of fNIRS analysis**

Fluctuations in the concentration of delta-oxygenated HbO2 were calculated from changes in detected light intensity according to the modified Beer-Lambert Law and the assumption of constant scattering ^[1]^. After data preprocessing (Details see supplemental materials), the changes in blood oxygen concentration in the ROI channels were calculated for statistical analysis. Data preprocessing was carried out after delta HbO2 signals were obtained and utilized the moving average filter at 2 s ^[2]^. Motion artifacts were subsequently eliminated using a processing technique based on moving standard deviation and cubic spline interpolation ^[3]^. By recognizing the sliding window standard deviation above a specific threshold, artifacts were identified and eliminated using cubic spline interpolation. The physiological signals were removed using the low-pass filter (0.1 Hz). The low frequency drift was removed by a high-pass filter (0.01 Hz) ^[4]^.

We used the data recorded in long-interval ANT to calculate brain activation. The overall level of task activation for each network was obtained by averaging the activation levels for all trials and all subjects by brain region. The data were extracted from a period of 1 s prior to and 9.1 s following the onset of the cue on each trial. According to Fan et al ^[5]^, the relative change in activation was calculated by subtracting the value between the two markers.

Resting-state functional connectivity was calculated to evaluate the effects of BCI training on brain functional activity. The standard ANT was used to analyze functional connectivity during ANT between ROIs. The coherence calculation was conducted using MATLAB scripts in order to reflect functional connectivity between bilateral posterior parietal and prefrontal cortices. Details see supplemental materials.

The GRaph thEoreTical Network Analysis (GRETNA) toolbox was used for graph theory analysis. Weighted and undirected networks were constructed based on coherence. The average interregional efficiency between every channel pair in each ROI is called local network efficiency, which measures the efficiency in transporting information for each ROI ^[6]^. Nodal efficiency and degree centrality are commonly used metrics to calculate local network efficiency ^[7]^.

**Calculation of coherence**

A Welch averaged, modified periodogram method ^[8]^ was used to calculate the squared coherence between each pair of channels. All connectivity matrices were Fisher’s z-transformed into a set of Gaussian distribution values ^[4]^. The mean z-score for each channel pair between ROIs was calculated for statistical analysis.

**GRaph thEoreTical Network Analysis**

We integrated metrics from the full threshold range (i.e., 0.1 to 0.4, with an interval of 0.05) to obtain the area under the curve (AUC) characterizing the brain network. Based on graph theory, the average interregional efficiency between every channel pair in each ROI is called local network efficiency, which measures the efficiency in transporting information for each ROI ^[6]^. Nodal efficiency and degree centrality are commonly used metrics to calculate local network efficiency ^[7]^.

Nodal efficiency is defined as the inverse of the harmonic mean of the shortest path length between the given node and all other nodes in the brain network ^[9]^.

$$E_{i}=\frac{1}{N-1}\sum_{j\in N, j\neq i} \frac{1}{D\left( i,j \right)}$$

where D (i, j) is the shortest path length between node i and node j, and N is the number of nodes in the network.

Degree centrality is a graph metric that assesses the importance of each node in a brain network,

evaluating the connectivity strength to every voxel ^[10]^.

$${DC}_{i}= \sum_{j=1}^{N} a_{ij}$$

The element a_ij_ of the adjacency matrix represents the connection or edge from node i to node j, which is 0 if no edge exists and nonzero for an edge with a weight a_ij_.

**Details of BCI training**

Video clips of people swimming were played on the screen by computer processing, and the subjects were guided to concentrate on their imagination in order to achieve and maintain a maximum uniform (40 r/min) running speed. Before commencing the experiment, the Link cotton was fully moistened with saline to facilitate good contact with the scalp and a robust EEG signal. The upper limb movement and muscle strength were set at a level of 0-1, while the training intensity was set at level 10. In this mode, the running speed related to the degree of involvement of motor imagery. The motor proportion was calculated by the ability to switch motor states in a single training session and the ability to maintain motor imagery. The higher the proportion, the more stimulation and the higher the training efficiency.

**Supplemental Figure**

**
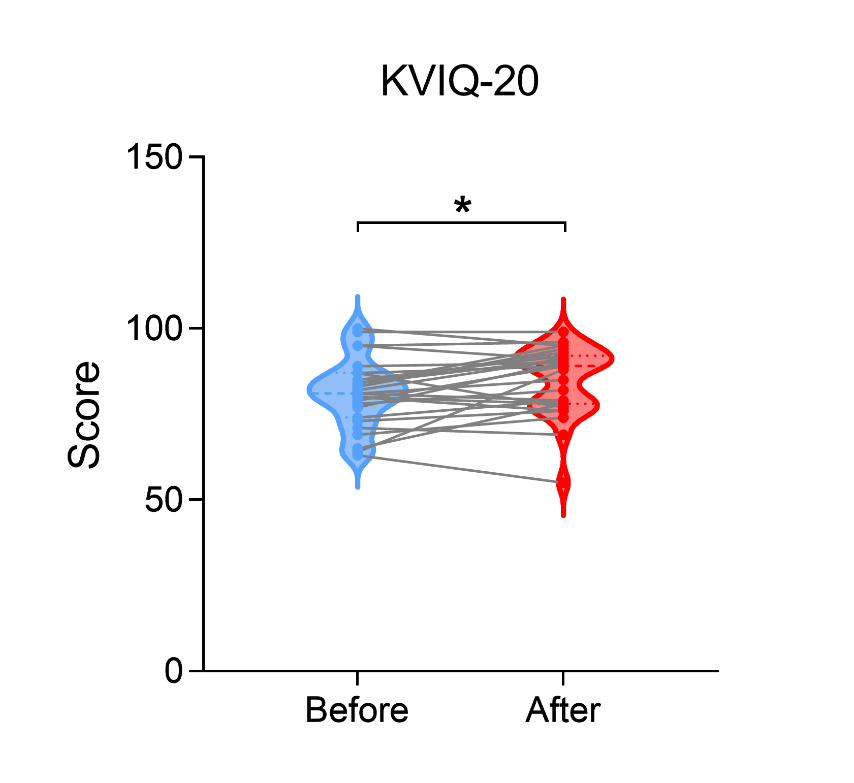
**

**Figure S1: The results of KVIQ-20.** The ability of motor imagery was significantly increased after BCI training. ^*^*P* < 0.05.

**Reference**

[1] SAKATANI K, YAMASHITA D, YAMANAKA T, ODA M, YAMASHITA Y, HOSHINO T, et al. Changes of cerebral blood oxygenation and optical pathlength during activation and deactivation in the prefrontal cortex measured by time-resolved near infrared spectroscopy [J]. Life Sci, 2006, 78(23): 2734-41.

[2] HUO C, XU G, LI Z, LV Z, LIU Q, LI W, et al. Limb linkage rehabilitation training-related changes in cortical activation and effective connectivity after stroke: A functional near-infrared spectroscopy study [J]. Sci Rep, 2019, 9(1).

[3] SCHOLKMANN F, SPICHTIG S, MUEHLEMANN T, WOLF M. How to detect and reduce movement artifacts in near-infrared imaging using moving standard deviation and spline interpolation [J]. Physiol Meas, 2010, 31(5): 649-62.

[4] ARUN K M, SMITHA K A, SYLAJA P N, KESAVADAS C. Identifying Resting-State Functional Connectivity Changes in the Motor Cortex Using fNIRS During Recovery from Stroke [J]. Brain Topogr, 2020, 33(6): 710-9.

[5] FAN J, MCCANDLISS B, FOSSELLA J, FLOMBAUM J, POSNER M. The activation of attentional networks [J]. Neuroimage, 2005, 26(2): 471-9.

[6] DING Q, ZHANG S, CHEN S, CHEN J, LI X, CHEN J, et al. The Effects of Intermittent Theta Burst Stimulation on Functional Brain Network Following Stroke: An Electroencephalography Study [J]. Front Neurosci, 2021, 15: 755709.

[7] ZOMORRODI R, LOHESWARAN G, PUSHPARAJ A, LIM L. Pulsed Near Infrared Transcranial and Intranasal Photobiomodulation Significantly Modulates Neural Oscillations: a pilot exploratory study [J]. Sci Rep, 2019, 9(1).

[8] WELCH. The Use of Fast Fourier Transform for the Estimation of Power Spectra: A Method Based on Time Aver. aging Over Short, Modified Periodograms [J]. 1967.

[9] ISMAIL L E, KARWOWSKI W. A Graph Theory-Based Modeling of Functional Brain Connectivity Based on EEG: A Systematic Review in the Context of Neuroergonomics [J]. IEEE Access, 2020, 8: 155103-35.

[10] ZUO X-N, EHMKE R, MENNES M, IMPERATI D, CASTELLANOS F X, SPORNS O, et al. Network Centrality in the Human Functional Connectome [J]. Cereb Cortex, 2012, 22(8): 1862-75.
